# *In Silico* Bioprospection of *Daniellia oliveri–*Based Products as Quorum Sensing Modulators of *Escherichia coli* SdiA

**DOI:** 10.1155/bri/7191508

**Published:** 2025-06-09

**Authors:** Yamkela Dweba, Christiana Eleojo Aruwa, Saheed Sabiu

**Affiliations:** Department of Biotechnology and Food Science, Faculty of Applied Sciences, Durban University of Technology, Durban 4000, South Africa

**Keywords:** *Daniellia oliveri*, *E.coli*, MD simulation, molecular docking, quorum sensing, SdiA

## Abstract

*Escherichia coli* is a common pathogen responsible for various gut-related infections, and it utilizes the SdiA-mediated quorum sensing (QS) system to regulate biofilm formation, other virulence factors, and pathogenicity. With rising antibiotic resistance, there is a pressing need to discover alternative QS inhibitors (QSIs) targeting SdiA. This study evaluated 239 phytochemicals from *Daniellia oliveri* as potential SdiA modulators using in silico techniques. Virtual screening identified four lead compounds (cadala-1(10),3,8-triene, carotenoid K, valencene, and β-sesquiphellandrene), with carotenoid K (−53.71 kcal/mol) exhibiting a higher binding free energy compared to the standard, azithromycin (−52.19 kcal/mol), following dynamics simulation. Notably, the SdiA-carotenoid K complex demonstrated enhanced thermodynamic stability with a root mean square deviation (RMSD) of 2.64 Å. All four leads, except carotenoid K, conformed to the Lipinski rule for selection of candidates that could be administered orally. Quantum chemical feature analyses using DFT/B3LYP showed that carotenoid K had the lowest HOMO-LUMO energy gap, high ionization energy, and electrophilicity index values, indicating its superior reactivity and stability. These properties suggest enhanced interactions with the SdiA active site compared to other investigated compounds. These observations highlight carotenoid K as a promising modulator of SdiA. However, further structural modification and validation through *in vitro* and *in vivo* studies are recommended.

## 1. Introduction

*Escherichia coli* is an extensively researched Gram negative bacterium for its role as a pathogen implicated in nosocomial infections. It forms part of the human normal microbiota and particularly inhabits the large intestines where it plays a huge role in maintaining gut health [1]. Although some *E. coli* strains are considered harmless and beneficial, some are known to cause various community-acquired and healthcare-associated infections such as urinary tract infections (UTIs), gastroenteritis (diarrhea), sepsis, wound infections, meningitis, and other soft tissue-related infections, especially in individuals with suppressed immune systems [2]. Its associated infections are considered a significant health concern on a global scale, as they pose a critical threat to public health. The threat they pose is on par with the threat posed by *Enterococcus faecium*, *Staphylococcus aureus*, *Klebsiella pneumoniae*, *Acinetobacter baumannii*, *Pseudomonas aeruginosa*, and *Enterobacter* spp. (ESKAPE) group of priority pathogens requiring new antibacterial interventions. The prevalence of drug resistance in *E. coli* has further made it difficult to treat associated infections over the years [3]. Reports confirm that various *E. coli* strains exhibit multidrug resistance (MDR) against the biocidal action of many conventional antibiotics such as cephalosporins, fluoroquinolones, and trimethoprim or sulfamethoxazole, alongside the ability to disseminate resistance genes to other bacteria [4, 5].

One of the ways that bacteria, including *E. coli*, use to successfully cause infections is through the formation of biofilms (aggregation of bacterial colonies within an extracellular matrix) that act as a shield from harsh environments while also limiting the uptake of antimicrobial drugs. Thus, biofilms contribute to antimicrobial resistance [6, 7]. The formed biofilm enhances bacterial growth, perpetuates antimicrobial resistance (AMR) and immune cell invasion, and becomes a hub for the exchange of genetic material [8]. There are reports that confirm that in *E. coli* there is a connection between diarrhea, virulence gene expression, and biofilm formation [9]. Hence, the focus now is on finding alternative ways of addressing AMR aided by biofilm formation via discovery and development of quorum sensing inhibitors (QSIs).

Quorum sensing (QS) has emerged as one of the most suitable targets for the attenuation of bacterial virulence and pathogenicity. Bacterial species employ QS to regulate genetic expression factors such as biofilm and toxins production, and drug resistance [10]. This process is dependent on the production, release, and detection of small signaling molecules called autoinducers. When inducer molecules reach a threshold concentration, population density increases, and inducer molecules bind to receptors to bring about a coordinated response that controls bacterial pathogenicity [11]. In *E. coli*, QS is controlled by three pathways, namely, autoinducer-2 (AI-2)- and SdiA mediated, and QseC/QseB two component QS systems. The SdiA-mediated QS takes precedence over the other systems because of its central and critical role in interspecies communication and regulation of virulence factors in members of the Enterobacteriaceae family [12, 13].

The SdiA protein is a homolog of LuxR that exists as an orphan transcriptional activator lacking an autoinducer (acyl-homoserine lactone, AHL) synthase counterpart. This means that the bacterium does not produce its own AHLs, but rather SdiA serves as a receptor for AHLs produced by other bacterial species [14]. This leads to the regulation of expression of various genes involved in biofilm formation, curli production, adhesion to epithelial cells, virulence, and motility, thus highlighting its significant role in *E. coli*'s pathogenicity and as a potential drug target for QS disruptors discovery [14]. Although SdiA is a LuxR-type homolog, it has a different trait where it can bind to the cell division operon, *ftsQP2*, independently of AHL binding. This suggests a role where this protein activates gene expression regardless of molecule signaling [15]. This further highlights the importance of this protein in *E. coli* gene expression and underscores its potential as a suitable drug target to control its virulence and pathogenicity [16].

In the search for alternative remedies to address AMR, plants have gained immense interest as sources of new potential antimicrobial therapeutics. This is guided by their centuries-old use in traditional medicine systems, rich content of bioactive compounds, and broad-spectrum activities. Hence, their use in current drugs (antimicrobials) discovery and development efforts to mitigate the growing trend of resistance to conventional antibiotics [17, 18]. Medicinal plants possess a plethora of bioactive metabolites such as alkaloids, saponins, tannins, phenolics, and flavonoids which demonstrate antibacterial and anticancer activities [19, 20]. *Daniellia oliveri (Rolfe)* Hutch. and Dalz is an example of a medicinal plant native to South America but also found in Africa [21]. The plant forms part of traditional medicine regimens because of its rich content of phytochemicals [22, 23].

The various parts of the *D. oliveri* tree possess numerous nutritional compositions [22]. In the *Daniellia* genera, *D. oliveri* is mostly exploited because of its economic value linked to the medicine industry. However, despite its great potential as a source of therapeutic chemical, it is still underutilized. Although medicinal plant derivatives such as those inherent in *D. oliveri* have been reported to possess QS modulating [24, 25] and antibacterial properties [26], there is still a lack of information on its anti-quorum sensing (AQS) activity. Hence, this study evaluated QS inhibitory potential of *D. oliveri*–derived phytochemicals against the SdiA receptor of *E. coli* using computational techniques [molecular docking, pharmacokinetics prediction, DFT analysis, and molecular dynamics (MD) simulation]. The use and integration of several *in silico* workflows provide key mechanistic insights at a molecular level and are efficacious in natural products high-throughput screening, prediction of lead molecules binding affinities and key interactions, pharmacokinetic attributes, and their activity validation in simulated solvated environments. Hence, computational tools advance the rate at which new drugs can be discovered and developed for various applications such as QS modulation [27].

## 2. Methodology

### 2.1. Ligands and SdiA Receptor Acquisition, Preparation, and Docking

The *E. coli* SdiA QS transcriptional regulator (sequence length 248) three-dimensional structure was obtained from the protein data bank (PDB ID: 4lgw) (https://www/rscb.org/pdb) in PDB format, and it was subsequently prepared using the Chimera 1.15 software [28]. The binding site was determined via Discovery Studio version 21.1.0 [29]. The ligands of interest (239 identified compounds of *D. oliveri*) were sourced from literature [26]. The 3D structures of most ligands were acquired from the PubChem database (https://pubchem.ncbi.nlm.nih.gov/) as structure data files (SDF), while those not available in the database were drawn using Marvin 18.30, 2018, ChemAxon (https://www.chemaxon.com). The conventional macrolide antibiotic, azithromycin, was used as a standard for the docking procedure [30]. Prior to docking, all ligands were optimized using the Open Babel tool version 2.3.2 found in the Python Prescription (PyRx) 0.8 Version and involved the addition of Gasteiger charges. Subsequently, the Vina tool plug-in on PyRx was employed to facilitate the molecular docking process [31]. After docking, the BIOVIA Discovery Studio software was used to visualize the interaction of ligands with the protein structure, as well as in assembling the complexes. The docking method was validated using the redocking method to eliminate any false positive results. This was done via lead compounds superimposition and cognate inhibitor re-docking at the co-crystallized SdiA binding domain. Thereafter, the obtained deviation value (≤ 2 Å) helped to determine the reliability of the docking protocol [32].

### 2.2. Drug-Likeness and ADME Features Prediction

The pharmacokinetic properties of the top performing compounds were evaluated via the SwissADME web server (https://swissadme.ch/index.php) to obtain a reliable forecast of the physicochemical parameters such as absorption, distribution, metabolism, excretion (ADME), and drug-likeness features of the lead compounds [33]. The Lipinski's rule of five (Ro5) was used as a filter for determination of drug-like compounds best suited for the development of oral medication [34].

### 2.3. MD Simulation

The MD simulation was conducted according to the methods described by Gonnet [35] and Ylilauri and Pentikäinen [36]. To summarize, the AMBER 18 software suite on Center for High Performance and Computing (CHPC) cluster (“HEAL1361” group-specific program) was used together with the FF18SD force field variant to run the simulation for 200 ns and characterize the operating systems. The atomic partial charges of ligands were computed using the ANTERCHAMBER tool in AMBER 18 software suit by implementing the general amber force field (GAFF) parameters and restrained electrostatic potential (RESP) methods. The systems were neutralized by the addition of hydrogen atoms, Na^+^, and Cl^−^ counter ions through the leap module tool. All the systems were placed in an orthorhombic box consisting of TIP3P water molecules, ensuring that all the systems are within the box. The SHAKE algorithm was applied to constrain fast moving hydrogen bonds in all simulated systems. Postdynamic data (root-mean-square deviation [RMSD], root mean square fluctuation [RMSF], radius of gyration [RoG], among others) processing was done via the CPPTRAJ program. The binding free energies of each simulated system for the 200 ns simulation period were calculated using the Molecular Mechanics/GB Surface Area (MMGBSA) method, which covered an average of 10,000 frames taken within a 200 ns simulation trajectory. The Discovery Studio software version 21.1.0 [29] was used to analyze and visualize each system's interactions from the snapshots generated as part of the postdynamic data. The Origin 6.0 analysis and graphing software were used to generate the data plots for the all the postdynamic data [37].

### 2.4. Quantum Chemical Calculations

The density functional theory (DFT) was employed with the aim of determining the molecular properties of the top performing compounds and in turn compute their electronic structure properties [38]. The Gaussian 16 software with the DFT/B3LYP/6-31G/+ basis set were used for the optimization of the compounds because of relatively superior results and lower computational costs [39]. The Gauss View version 6.0.16 software was used to visualize the results to show the frontier highest occupied molecular orbital (HOMO) and lowest unoccupied molecular orbital (LUMO) as well as the generation of the electrostatic potential (ESP) and the visualization of ESP surfaces to evaluate the electrostatic induced weak interaction [40]. The conceptual density functional theory (CDFT) was used as a basis to calculate the chemical descriptors (ionization energy, energy gap, softness, hardness, and global electrophilicity, among others) that elaborate on the molecular properties of chemicals; these were calculated using the energies of the frontier molecular orbitals (HOMO and LUMO) as a basis. These calculations were performed in line with Koopmans' theorem and Parr-Pearson interpretation of DFT [41].

## 3. Results and Discussion

### 3.1. Molecular Docking Outcomes

**Figure 1:**
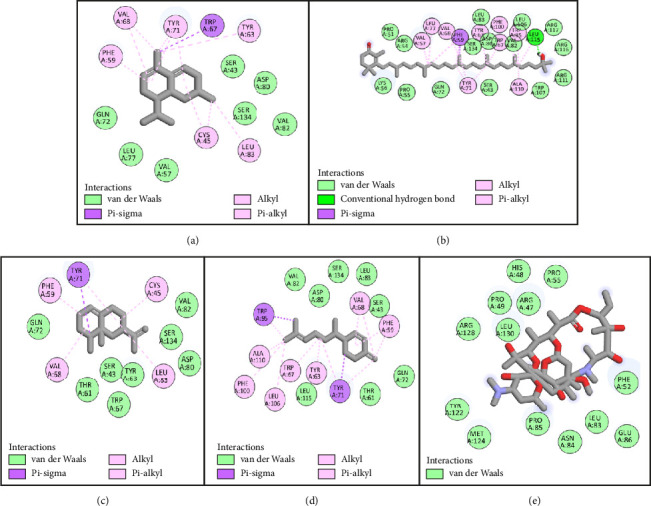
2D docking interactions of top four *D. oliveri* compounds and standard. (a) Cadala-1(10),3,8-triene; (b) carotenoid K; (c) valencene; (d) β-sesquiphellandrene; (e) azithromycin.

Following molecular docking of *D. oliveri* compounds at the active site of *E. coli* SdiA, the top four best performing compounds were identified based on their docking scores and best pose within the target site. These selection criteria are a preliminary measure of their binding fitness toward the protein and translates to how strong and favorable their predicted interaction may be [42]. A higher negative docking score translates to a stronger binding affinity between the protein and ligand, as well as more favorable interactions that are potential indicators of a good candidate for further evaluation [43].  Table 1 details the docking score/binding affinity of the *D. oliveri* constituents in terms of docking scores (kcal/mol), as well as their molecular interactions with the different amino acid residues (Figures 1(a)–1(e)). The docking scores of the top four *D. oliveri* compounds ranged between −9.4 and −10.2 kcal/mol; cadala-1(10),3,8-triene (−10.2 kcal/mol), carotenoid K (−10.1 kcal/mol), valencene (−9.6 kcal/mol), and β-sesquiphellandrene (−9.4 kcal/mol), exhibiting higher negative docking scores and higher number of interactions compared to the standard, azithromycin (−6.0 kcal/mol). In contrast, a study of 5-hydroxymethylfurfural (5-HMF) and its derivatives bound to *E. coli* SdiA reported a range of lower negative docking scores (−5.7 to −4.9 kcal/mol) compared to ours [44]. This indicates a reduced fitness and SdiA target inhibition of 5-HMF and its derivatives relative to our lead compounds. This observation of higher negative docking scores suggests a tighter binding via the formation of more stable interactions with SdiA active site residues compared to the standard. Hence, the leads could be more effective inhibitors of SdiA [45]. These findings are in line with a study by S'thebe et al. [46] where plant-derived compounds (phylloquinone, linoleic acid) displayed higher negative docking scores (−9.4 to −8.5 kcal/mol) relative to the standard at −6.4 kcal/mol, against *Pseudomonas aeruginosa* LasR protein.

Again, the ligands interacted with SdiA's binding pocket via Pi- and van der Waals interactions (Table 1, Figures 1(a)–1(e)), which hint at the hydrophobic nature of the active site owing to the presence of nonpolar hydrophobic amino acid residues (phenylalanine, tryptophan) [47]. This may also indicate that ligand binding is facilitated through hydrophobic interactions which serve a crucial role in aiding ligand adaptation at the binding site. They further provide the needed flexibility amid any conformational changes that might occur within the protein [48]. Although weaker than the hydrogen bonds observed in carotenoid K, the presence of hydrophobic bonds bring about great stability within the protein. The SdiA protein also retains critical amino acid residues that facilitate AHL binding. These include tyrosine 63 (Tyr63), tryptophan 67 (Trp67), serine 79 (Ser79), aspartic acid 80 (Asp80), lysine175 (Lys175), and arginine 182 (Arg182). Although only carotenoid K formed a hydrogen bond with Leu115, all leads formed hydrophobic interactions with Tyr63, Trp67, Val68, Tyr71, Phe100, and Ala110. In consonance with our findings, Singh et al. [44] also reported that hydrophobic interactions were made with Tyr63, Trp67, Val68, Tyr71, Phe100, Ala110, Leu115, and Arg116 at the SdiA active site, upon binding of 5-HML and its derivative compounds. The ability of our top four *D. oliveri* compounds to interact with critical amino acids may be responsible for the obstruction of substrate access through competitive or noncompetitive inhibition of AHL binding. This could result in disruption of the SdiA QS pathway via virulence genes expression blockage [49].

**Figure 2:**
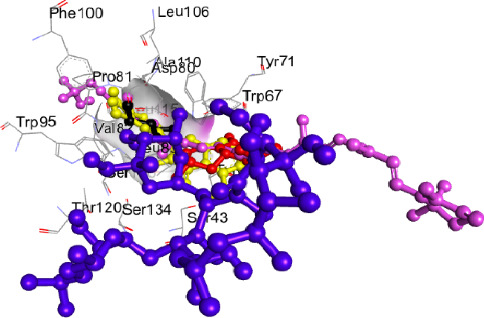
Validation of the docking technique via superimposition of lead compounds and standard on the co-crystal structure of *E. coli* SdiA (RMSD: 0.5 Å). Cadala-1(10),3,8-triene (red); carotenoid K (pink); glycerol (native ligand, black); azithromycin (purple).

Following the derivation of docking scores, the docking method was validated to confirm the reliability, accuracy, and reproducibility of the results obtained [50]. The redocking process (superimposition of ligands at SdiA active site) showed that the most active compounds assumed a similar docking process with an RMSD of 0.5 Å. This confirmed that docking was accurately done. The RMSD value of < 1 suggested the same binding orientation relative to its starting point in the final complex after redocking (Figure 2) [32].

### 3.2. Pharmacokinetic Properties Assessment

In the search for promising drug candidates, it is crucial to determine their pharmacokinetic properties to avoid high failure rates recorded at the preclinical and clinical trial phases of drug development. Evaluating the pharmacokinetics, drug likeness, and toxicity profiles of the lead compounds serves as an initial indicator of the compound's in vivo medicinal friendliness [51, 52]. Interestingly, three of the four lead compounds, namely, cadala-1(10),3,8-triene, valencene, and *β*-sesquiphellandrene passed the LRo5 with at least one violation. Carotenoid K (molar weight 568.9 g/mol; Log*p* of 10) and azithromycin (molar weight 748.9 g/mol; 14 H-bond acceptors) had two violations each. Thus, the latter two ligands failed to satisfy the LRo5 (Table 2). Nevertheless, all lead compounds remain potentially viable even beyond the LRo5 [53] for further exploration as QSIs targeting SdiA. Adeosun et al. [54] also reported LRo5 violation for lead ligands, phytol (LogP), and genistin (H-bond donor > 5), which were identified as lead *K. pneumoniae* SdiA antagonists. The Ro5 is a standard measure for selecting orally active drugs, where compounds should not be in violation of more than one of the rules [34]. The oral bioavailability of lead *D. oliveri* compounds depicts their potential to reach the target site while exerting maximal therapeutic effects. In the case of carotenoid K, the option to look beyond the Ro5 still exists for potential bioactive drug candidates that maintain other good pharmacokinetic profiles such as high permeability, unique structure and mechanisms of action, and good metabolism [55]. Such cases are common with structurally modified antibiotics (azithromycin, vancomycin, or aminoglycosides), peptides, central nervous system (CNS) drugs, and even natural compounds [56]. Hence, carotenoid K still stands as a good candidate for drug development regardless of its violations of the Ro5.

Cadala-1(10),3,8 triene, valencene, and β-sesquiphellandrene exhibited high solubility in water (bioavailability score of 0.55 each), which suggested that more than half (55%) of these compounds would reach systemic circulation intact in their active form and exert therapeutic effects when administered [57]. On the other hand, both carotenoid K and azithromycin had a bioavailability score of 0.17 (low bioavailability, poor absorption, and limited effectiveness). This meant that up to 83% of the “drug” could go through first-pass metabolism and be wasted/excreted [42]. Circumventing this may mean the use of the compound in higher doses but with mindfulness of their toxicity to ensure they reach maximum therapeutic levels. This is applicable if the chosen route of administration remains oral [58]. Gastrointestinal (GIT) absorption plays a huge role in determining the bioavailability of a drug, and high GIT absorption results in maximum bioavailability and vice-versa. However, findings showed that all the compounds, including the standard, had low GI absorption. As such, all the compounds may be difficult to dissolve in GI fluids, insoluble, or exhibit poor GI membrane permeability, which could then lead to reduced bioavailability [59]. According to Millar et al. [60], modifying the drug formulation, choosing alternative routes of administration (intravenous, intranasal), or novel drug delivery systems could aid in circumventing this challenge. Administration routes that provide rapid systemic effects and increased bioavailability relative to oral delivery aid in bypassing the first-pass metabolism [61].

### 3.3. Structural Dynamics of Ligand-Protein Complexed Systems

#### 3.3.1. Energy Components Analysis

The postdynamic data (energy components) calculated using the MMGBSA module are presented as the thermodynamic binding free energy (Δ*G*_bind_) (Table 3). The Δ*G*_bind_ is an important descriptor in dynamics studies. It describes the change in free energy because of the interactions between a ligand and its receptor. It is crucial in elucidating the strength and stability of a protein-ligand interaction and may also give an insight into the efficacy of promising drug candidates [62]. In essence, the higher the negative Δ*G*_bind_ value, the better the ligand's affinity for the protein and thermodynamically favorable interactions [63, 64]. The study found that the top performing compounds, except for carotenoid K, had lower binding free energies compared to the standard, azithromycin. β-sesquiphellandrene-, cadala-1(10),3,8-triene-, and valencene-SdiA complexes had binding free energy scores of −33.44 kcal/mol, −29.35 kcal/mol, and −34.59 kcal/mol, respectively, but which were lower than those observed for carotenoid K (−53.71 kcal/mol) and azithromycin (−52.19 kcal/mol) systems (Table 3). Comparable Δ*G*_bind_ values similar to that observed for carotenoid K have been reported for *K. pneumoniae* SdiA lead inhibitors, WAY-310622 (−53.19 kcal/mol) and WAY-660842 (−53.91 kcal/mol) [65]. In contrast, Vargas et al. [66] recorded a weaker binding energy value of −50.72 kcal/mol for a nonsteroidal anti-inflammatory drug, morniflumate, bound to *E. coli* SdiA. Our findings thus suggest the greater potential of carotenoid K as an inhibitor of *E. coli* SdiA relative other compounds, including the standard. In a nutshell, the carotenoid K-SdiA complex showed higher affinity for the SdiA-binding domain. Strong bonds ensure stability of leads at the target binding site with little or negligible dissociation events. This could translate to having an increased therapeutic effect [64, 67]. This makes carotenoid K the most promising drug candidate among the other top performing *D. oliveri* compounds. The reduced thermodynamic stability and affinity of other compounds for SdiA may diminish their biological activity (AQS) [45]. Nonetheless, these results are relatively aligned with docking outcomes that emphasize carotenoid K as the lead compound with one H-bond, higher negative docking score, and binding free energy, relative to the standard.

### 3.4. Thermodynamic Stability, Compactness, and Flexibility of Leads Bound to *E. coli* SdiA

**Figure 3:**
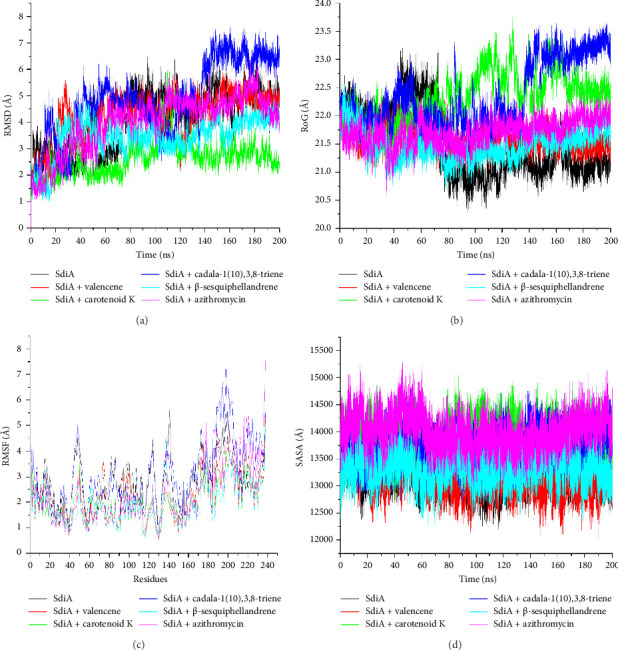
Comparative post-MD simulation plots of the alpha-carbon, the top four compounds, and azithromycin against the *E. coli* SdiA active site over a 200-ns MD simulation period. (a) Root mean squared deviation (RMSD) plots; (b) radius of gyration; (c) root mean square deviation (RMSF); (d) solvent accessible surface area (SASA).

**Figure 4:**
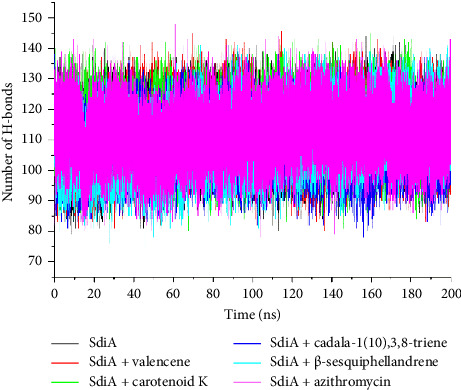
Time evolution of the number of intramolecular hydrogen bonds formed following binding of the top four *D. oliveri* compounds and azithromycin at the *E. coli* SdiA active site over a 200-ns MD simulation period.

MD simulation is an important in silico validation technique used in drug development to provide invaluable information into molecular interactions, stability, and dynamic behavior of potential drug candidates [68]. It reveals how drugs interact with their biological targets, that is, interactions, strength, and binding effects on the structure of the target [69]. The dynamics simulation complements and further validates outcomes from molecular docking by providing deeper insights into the dynamics of bound protein-ligand systems. The binding free energy parameter is further supported by the evaluation of other key descriptors of thermodynamic stability such as RMSD, RoG, solvent accessible surface area (SASA) (Figures 3(a)–3(d)), and intramolecular hydrogen (H) bonds (Figure 4), as well as the mean measure of these same parameters (Table 4).

The RMSD measures bound systems' deviation over time in relation to the unbound structure. It also defines stability and assesses the conformational changes in the protein structure [70]. The lower values of RMSD relate to a more equilibrated system relative to the reference structure during the simulation window. Usually, values less than 3 Å are within acceptable limits and represent good stability of the complexed system [71]. The derived RMSD data over the 200 ns simulation are represented in Figures 3(a)–3(d), and average postdynamic values are given in Table 4. Following the initial convergence at 10 ns and at 20 ns, the SdiA-cadala-1(10),3,8-triene complex showed minimal fluctuations but thereafter showed significant fluctuations. A similar observation was made for the apo structure of SdiA (stable from 0–70 ns, then following higher deviated trajectory after the 70 ns point). The SdiA-carotenoid K and SdiA-β-sesquiphellandrene complexes, regardless of having significant fluctuations, showed the most equilibrated fluctuation patterns. This suggested superior stability over the other systems. All other systems except the SdiA-carotenoid K complex had RSMD values beyond the benchmark of 3 Å. A study targeted at showing the affinity of several exogenous autoinducer compounds against SdiA showed that only the AHLs, 3-oxo-C8-HSL (2.71 Å), and 3-oxo-C10-HSL (2.31 Å) had RMSD values comparable to carotenoid K, while other AHL molecules bound to *K. pneumoniae* SdiA (3.29–5.54 Å) and the apo-protein (3.74 Å) had above 3 Å RMSDs [72]. Overall, our findings showed that carotenoid K had high conformational rigidity and enhanced specificity for SdiA, and this is advantageous for a potential drug candidate [73]. The next relatively stable system was the SdiA+β-sesquiphellandrene complex (mean RMSD of 3.44 Å). Higher deviations may be attributed to the significant fluctuations during the simulation window. These findings are consistent with the literature where greater stability is proportional to lower RMSD values [74]. The fact that these compounds outperform the standard is very much suggestive of their high prospect as lead inhibitors of SdiA.

The RoG descriptor assesses stability from an angle of overall structural compactness. Low RoG values are indicative of a highly compact structure with enhanced stability [75]. The RoG plot between 0 and 60 ns revealed minimal fluctuations in compactness in all systems. However, beyond this point, significant fluctuations could be observed, particularly for SdiA-carotenoid K and SdiA-cadala-1(10),3,8-triene systems (Figure 3(b)). This observation was further supported by their high average RoG values of 22.34 Å and 22.33 Å, respectively (Table 4). All other lead compounds systems, including the unbound SdiA (21.50 Å) and azithromycin (21.71 Å), were well equilibrated from 60 ns. These observations could be linked to initial adaptation of systems in response to binding within the first 60 ns in a bid to stabilize the protein. However, during the simulation, the ligands established new interactions leading to the protein adapting to newer conformations that ultimately resulted in reduced compactness [76]. These dynamic shifts because of ligand binding determines critical structural rearrangements such as protein folding and unfolding [77]. Contrary to the results obtained from the RMSD plots, carotenoid K exhibited the highest average RoG value, indicating its lesser compact nature and relative instability compared to the other systems. Certain factors such as protein topology, size, length, nature of the active site, and the types of amino acid have been shown to influence protein folding and unfolding, and thus may be responsible for the observed RoG patterns in this study [78].

The RMSF quantifies the thermodynamic flexibility of the amino acid residues within the protein structure over time. Lower RMSF values relate to greater stability, reduced flexibility, the formation of stronger bonds, and better affinity for the protein [79]. In this study, the RMSF plots revealed a similar fluctuation pattern among the SdiA-ligand complexes. The level of fluctuations varied from one system to the other, resulting in variations in the observed average RMSF scores (Table 4). The major fluctuations were observed between residues 40 and 50, 120 and 130, 130 and 150, 170 and 180, and 190 and 210. The most stable region was between R60 and –R110, which contains SdiA key amino acid residues (Figure 3(c)). The binding of carotenoid K, valencene, β-sesquiphellandrene, and the standard resulted in much lower fluctuations compared to those observed for the apo structure and SdiA-cadala-1(10),3,8-triene. This finding hints at possible alterations to SdiA linked to the binding of compounds [80]. On the other hand, the prominent fluctuations of SdiA suggest a more dynamic and less constrained nature of the protein which allows proteins to freely undergo the necessary structural changes required to establish interactions with many ligands [81]. However, high fluctuations also equate with high instability that could limit the ability of the protein to maintain a more functional state [82]. Mean RMSF values were within the acceptable limit of 3 Å. With the exception of cadala-1(10),3,8-triene (3.07 Å), all other lead complexes had lower mean RMSF scores relative to the apoprotein and standard. Our findings implied that with the exception of cadala-1(10),3,8-triene and the unbound SdiA (2.77 Å), all other lead compounds formed stronger interactions at the SdiA active site. Adeosun et al. [54] also showed low to relatively high RMSF scores ranging from 1.17 Å (sabinene) to 3.74 Å (camphene) for selected terpenes and flavonoids used in the study. Again, from the RMSF plot, there are regions that correspond to the key amino acids: Tyr63, Trp67, Ser79, Asp80, Arg182, and Lys175. These regions exhibited very low fluctuations, thus suggesting the stability of the active site residues. This finding buttresses the better affinity of these compounds (especially carotenoid K and β-sesquiphellandrene) as inhibitors of the SdiA target.

The SASA is another important parameter that provides information on the interactions between a protein and its external environment [83]. It also accounts for alterations in surface area during a simulation [84]. Low SASA values indicate a more structurally stable complex and decrease in surface area. In this study, minimal fluctuations were observed with the unbound SdiA (13,293 Å), valencene (13,168 Å), and β-sesquiphellandrene (13,321 Å) systems while carotenoid K, cadala-1(10),3,8-triene, and azithromycin complexes exhibited higher fluctuations, as depicted in the SASA plot (Figure 3(d)). Carotenoid K, cadala-1(10),3,8-triene, and azithromycin systems had SASA values of 13,893 Å, 13,700 Å, and 14,009 Å, respectively (Table 4). The fact that valencene and β-sesquiphellandrene exhibited SASA values that were closely related to that of the apoprotein but less than that of the standard showed that they effectively decrease the surface area of the protein by occupying the spaces that were previously exposed to the external solvated environment. The binding of carotenoid K, cadala-1(10),3,8-triene, and azithromycin led to a significant increase in SdiA surface area, which compromises the stability of the complex. However, this may reflect the conformational changes that took place while the protein was configuring itself to the best conformation needed to establish a stable state [83].

Intramolecular H bonds are vital in the stabilization of various biomolecules, including proteins. They influence stability and conformation, and contribute to the binding free energies of the systems [85]. A stable number of H bonds were formed which fluctuated between 80 and 145 (Figure 4). This observation is supported by the consistent mean number of H bonds across all systems. Unbound SdiA, SdiA-valencene, SdiA-carotenoid K, SdiA-cadala-1(10),3,8-triene, SdiA-β-sesquiphellandrene, and SdiA-azithromycin had average H bonds of 111.50, 110.23, 108.66, 113.51, 112.34, and 112.17, respectively. The formation of a comparable number of H bonds suggests that the structural integrity of SdiA is retained for the stabilization of the protein structure. Majewski et al. [89] stated that the presence of H bonds is expected in protein-ligand systems, as they maintain the structural framework of the protein which remains unchanged regardless of ligand binding. Again, the consistency of the formed H bonds showed that the impact of ligands binding is minimal on the protein structure, thereby maintaining complex stability [87]. Murugesan et al. [88] also confirmed that the formation of a steady number of H bonds by inhibitor molecules of the SARS-CoV-2 main protease M^pro^like astragalin, pectolinarin, and amritoside resulted in stronger interactions (high *Δ*Gbind) with the active site of the protein such that they did not significantly alter the H bond network.

### 3.5. Quantum Chemical Reactivity Attributes

**Figure 5:**
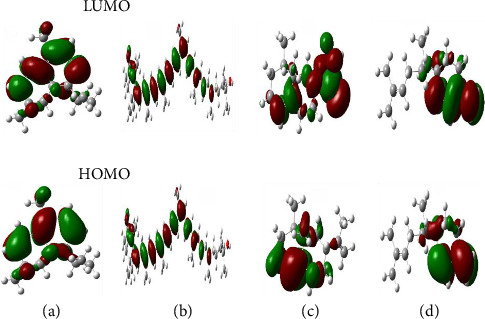
Frontier orbitals of the top four *D. oliveri* compounds. (a) Cadala-1(10),3,8-triene; (b) carotenoid K; (c) valencene; (d) β-sesquiphellandrene.

**Figure 6:**
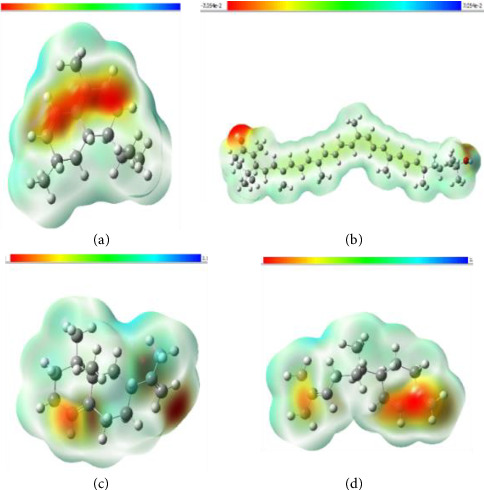
Electrostatic potential (ESP) mapped molecular surface of top four *D. oliveri* compounds. (a) Cadala-1(10),3,8-triene; (b) carotenoid K; (c) valencene; (d) β-sesquiphellandrene.

The DFT analysis was performed for the most promising *D. oliveri* compounds to evaluate their molecular properties. Quantum chemical features are crucial in determining compounds' stability, reactivity, and drug efficacy. They also aid in understanding their interaction with biological targets. These calculations are based on the HOMO and LUMO concepts that elucidate the chemical reactivity and binding properties of a compound [89, 90]. The HOMO-LUMO model is based on both high and low energy orbitals, and its relevance lies in the difference between their energy levels which serve as the key pointer to the stability and reactivity properties of lead bioactive molecules. These “indicator” features are integral to applications such as drug discovery (to comprehend molecule reactivity and interaction with a biological drug target) and quantum chemistry (to computationally model intramolecular charge and energy transfer interactions) [91]. Within the scope of this study, the model is captured for drug discovery application. The HOMO orbital elaborates more on the tendency of a compound to donate electrons, while the LUMO orbital sheds light on the electron withdrawing tendency of a compound [92]. The energy gap between HOMO and LUMO energies (Figure 5) aids in determining whether a system is more chemically reactive and also touches on the molecule's stability [93]. As such, a smaller energy gap indicates more reactivity as less energy is required for the molecule to donate or accept electrons, and the reverse is the case with larger energy gaps [92]. The quantum features of the top performing *D. oliveri* compounds were depicted in Table 5, and Figures 5 and 6. Of all the leads, carotenoid K had the lowest energy gap, followed by cadala-1(10),3,8-triene. This finding suggests that they have increased reactivity and less stability compared to other compounds. This feature is beneficial for forming strong interactions (covalent and H bonds) with the active site of SdiA. Their low chemical hardness and high softness further attests to their reactive nature [40]. Higher reactivity molecules could impact on Δ*G*_bind_ by disrupting the bound complex. Contrary to this, carotenoid K exhibited the highest negative Δ*G*_bind_ value among the screened compounds which denotes its high stability and suitability for the protein. These findings correlate with those of Ma et al. [40] where amentoflavone had the lowest energy gap and high reactivity but still had the highest Δ*G*_bind_ against SARS-CoV-2 main protease.

In addition, a low ionization energy meant that the compound's electrons are loosely bound, thus making it more reactive. High electron affinity values suggest higher electron withdrawing potential and chemical stability [94]. Valencene and β-sesquiphellandrene exhibited the lowest ionization energy and superior electron affinity values. This finding is consistent with their HOMO-LUMO energy gaps. Contrary to this, carotenoid K had the highest ionization energy, suggesting its electrons are tightly bound, and it is relatively stable. The electronegativity and chemical potential evaluate the ability of an atom or functional group to exchange electrons with its environment [95]. With regard to this, carotenoid K and cadala-1(10),3,8-triene had the highest electronegativity values and hence are good electron acceptors and stable compounds capable of forming strong bonds. This observation is in line with the observed ionization energy and electron affinity values for these compounds. The electrophilicity index (EI) defines the interaction between electrophiles and nucleophiles such that a high EI depicts a strong electrophile while a nucleophilic molecule exhibits lower EI [96, 97]. Again, carotenoid K and cadala-1(10),3,8-triene exhibited the highest EI values. In addition, the ESP regions (Figure 6) colored in blue promote compound electrophilicity, while yellow areas are nucleophilic, and green regions exhibit zero potential [98]. The mapped surfaces show the best sites identified for key inter- and intramolecule interactions to take place [99]. These findings seem to suggest that carotenoid K and cadala-1(10),3,8-triene may have decreased therapeutic efficacy because of low stability [100]. However, other descriptors infer a beneficial outcome in the context of therapeutic efficacy. For instance, the high EI translates to high reactivity of these compounds. Consequently, this would likely enhance their effectiveness in targeting SdiA. This is a common occurrence in covalent inhibitors that alter target proteins irreversibly, thereby exhibiting high therapeutic efficacy because of their ability to form stable interactions with their targets [101]. Hence, the study findings indicate carotenoid K as the most stable and reactive molecule of the four *D. oliveri* leads, suggesting its enhanced potential as an inhibitor or modulator of the SdiA QSS. Lastly, it is worth noting here that some of the potential aftereffects of *E. coli* SdiA inhibition by the identified *D. oliveri* leads (following structural modifications, and in vitro and in vivo validations) could include downregulated expression of *E. coli* fimbriae and motility genes, and disruption of interspecies sensing (communication) functions. Likewise, resistance regulation through downregulation of adhesion proteins or genes is an added favorable impact, as it could affect the ability of *E. coli* to infect and colonize efficiently [102].

## 4. Conclusion

The study identified lead *D. oliveri* compounds (carotenoid K, cadala-1(10),3,8-triene, valencene, and β-sesquiphellandrene) with potential to effectively bind the *E. coli* SdiA active site. These compounds exhibited the highest binding affinity for SdiA, and carotenoid K showed the best and strongest binding interactions with SdiA. All top-ranking compounds maintained key interactions with SdiA key amino acid residues that are critical to binding. This hints at their potential to effectively modulate the function of SdiA. Carotenoid K exhibited a higher negative Δ*G*_bind_ compared to other leads and the standard, azithromycin, thus highlighting its potential as an inhibitor of SdiA. All *D. oliveri* lead compounds exhibited low fluctuations at the regions corresponding to the key amino acid residues and formed stable bonds. Quantum chemical calculations revealed carotenoid K as the most suitable for further investigation, despite its lack of adherence to the LRo5 which only applies to orally administered drugs. This finding does not presuppose that carotenoid K might fall short in terms of efficacy, especially if another route of administration is used. Overall, findings indicate that carotenoid K is the most probable drug candidate for QSI development against *E. coli* SdiA. However, further structural modification and validation experiments (in vitro, in vivo) on carotenoid K and other leads is recommended to enhance their QS inhibiting activity.

## Figures and Tables

**Table 1:** Binding fitness and bonds formed between the top four compounds, standard, and *E. coli* SdiA.

Target	Ligand (CID)	Docking score (kcal/mol)	Total bonds	Conventional hydrogen bonds	Other significant bonds
*E. coli* (SdiA)	Cadala-1(10),3,8-triene (593,889)	−10.2	14	—	6 alkyl and pi-alkyl (Cys45, Phe59, Tyr63, Val68, Tyr71, and Leu83), 1 pi-sigma (Trp67), 7 van der Waals
	Carotenoid K (14730338)	−10.1	26	1 (Leu115)	9 alkyl and pi-alkyl, 1 pi-sigma (Phe59), and 5 van der Waals
	Valencene (9855795)	−9.6	13	—	4 alkyl and pi-alkyl (Cys45, Phe59, Val68, and Leu83), 1 pi-sigma (Tyr71), 8 van der Waals
	β-sesquiphellandrene (519764)	−9.4	17	—	7 alkyl and pi-alkyl (Phe59, Tyr63, Trp67, Val68, Leu106, Phe100, and Alal110), 2 pi-sigma (Tyr71, Trp95), and 8 van der Waals
	Azithromycin (447043)	−6.0	13	—	13 van der Waals

**Table 2:** Pharmacokinetic properties of the top four *D. oliveri* compounds interacting with *E. coli* SdiA.

Ligand (CID)	Mol. w. (≤ 500 g/mol)	HBD (≤ 5)	HBA (≤ 10)	Lipophilicity (Log*p* ≤ 5)	Bioavailability (water solubility)	GIT absorption	BBB permeability	Lipinski violations
Cadala-1(10),3,8-triene (593,889)	202.3	0	0	3.95	0.55	Low	No	Yes; 1 violation
Carotenoid K (14730338)	568.9	1	2	10	0.17	Low	No	No; 2 violations
Valencene (9855795)	204.3	0	0	4.41	0.55	Low	No	Yes; 1 violation
β-Sesquiphellandrene (519764)	204.4	0	0	4.56	0.55	Low	No	Yes; 1 violation
Azithromycin (447043)	748.9	5	14	2.02	0.17	Low	No	No; 2 violations

Abbreviations: BBB = blood–brain barrier; GIT = gastrointestinal tract; HBA = hydrogen bond acceptors; HBD = hydrogen bond donors; Log*p* = lartition coeMcient; Mol. w = molecular weight.

**Table 3:** Energy components (kcal/mol) of lead compounds and control against *E. coli* SdiA.

Systems	Δ*E*_vdW_	Δ*E*_elec_	Δ*G*_gas_	Δ*G*_solv_	Δ*G*_bind_
SdiA + β-sesquiphellandrene	−38.1 ± 2.17	−5.21 ± 1.44	−43.38 ± 2.91	9.94 ± 1.16	−33.44 ± 2.57
SdiA + Cadala-1(10),3,8-triene	−33.46 ± 1.78	−4.63 ± 1.34	−38.09 ± 2.39	8.74 ± 0.99	−29.35 ± 2.05
SdiA + carotenoid K	−67.11 ± 4.00	−15.28 ± 7.22	−82.39 ± 8.57	28.68 ± 6.47	**−53.71 ± 4.01**
SdiA + valencene	−38.62 ± 1.83	−2.37 ± 1.37	−40.99 ± 2.34	6.40 ± 1.03	−34.59 ± 2.18
SdiA + azithromycin	−33.75 ± 3.65	−93.41 ± 12.77	−147.93 ± 13.20	95.73 ± 12.33	−52.19 ± 5.73

*Note:* Bold values highlight the compound with the highest binding free energy and affinity for *E. coli* SdiA.

Abbreviations: Δ*E*_elec_ = electrostatic energy; Δ*E*_gas_ = gas phase free energy; Δ*G*_solv_ = solvation free energy; Δ*G*_bind_ = total binding free energy; Δ*E*_vdW_ = van der Waals energy.

**Table 4:** Average RMSD, RoG, RMSF, SASA, and intramolecular hydrogen bond number values of the top four compounds and controls following a 200-ns simulation at the *E. coli.* SdiA active site.

Systems	Number of H bonds	RMSD (Å)	RMSF (Å)	RoG (Å)	SASA (Å)
SdiA	111.50 ± 7.54	4.09 ± 1.09	2.77 ± 0.97	21.50 ± 0.59	13,293.25 ± 278.84
SdiA + Valencene	110.23 ± 7.94	4.23 ± 0.86	2.17 ± 0.87	21.60 ± 0.22	13,168.85 ± 289.75
SdiA + Carotenoid K	108.66 ± 7.24	2.64 ± 0.69	2.04 ± 0.77	22.34 ± 0.41	13,893.88 ± 274.07
SdiA + Cadala-1(10),3,8-triene	113.51 ± 7.46	4.82 ± 1.41	3.07 ± 1.26	22.33 ± 0.58	13,700.20 ± 275.93
SdiA + β-sesquiphellandrene	112.34 ± 7.71	3.44 ± 0.72	1.94 ± 0.81	21.48 ± 0.24	13,321.40 ± 252.44
SdiA + Azithromycin	112.17 ± 7.66	4.00 ± 1.04	2.23 ± 1.20	21.71 ± 0.22	14,009.40 ± 287.48

**Table 5:** The conceptual density functional theory (CDFT) of the top four *D. oliveri* compounds.

Descriptor	Cadala-1(10),3,8-triene	Carotenoid K	Valencene	β-Sesquiphellandrene
LUMO	−0.854	−2.488	0.799	−0.361
HOMO	−5.032	−4.496	−6.157	−5.815
Energy gap	4.178	2.008	6.957	5.453
Ionization energy	0.854	2.488	−0.799	0.361
Electron affinity	5.032	4.496	6.157	5.815
Hardness	2.089	1.004	3.478	2.727
Softness	0.479	0.996	0.287	0.367
Electronegativity	2.943	3.492	2.679	1.050
Chemical potential	−2.943	−3.492	−2.679	−1.050
Global electrophilicity	2.073	6.072	1.032	0.202

## Data Availability

The data that support the findings of this study are available on request from the corresponding author.
